# Individual variation is the key to the development of a vaccine against S*taphylococcus aureus*: a comparative study between mice lineages

**DOI:** 10.1590/1414-431X20186773

**Published:** 2018-03-26

**Authors:** D.P. dos Santos, I.P.R. Muniz, A.F. Queiroz, I.S. Pereira, M.P.A. Souza, L.J. Lima, L.R.O. Sousa, I.S. Ribeiro, M.P.L. Galantini, L.M. Marques, T.B. Figueiredo, R.A.A. da Silva

**Affiliations:** Instituto Multidisciplinar em Saúde, Universidade Federal da Bahia, Vitória da Conquista, BA, Brasil

**Keywords:** Immunization, Staphylococcus aureus, Mice, Air pouch, Antibodies

## Abstract

Bacterial infections occur worldwide and are a major public health problem. Among pathogens, *Staphylococcus aureus* is the main causative agent of bacterial diseases in the world. This study aimed to evaluate which components of the immune system could act protectively against a *S. aureus* infection in intradermally immunized mice. C57BL/6 and A/j mice were immunized intradermally with *S. aureus* inactivated by heat and then challenged with viable strains in an air pouch model. At 6, 12, and 24 h after the challenge, euthanasia was performed, and the cellular profile of the inflammatory infiltrate, cytokines, and the bacterial load were evaluated in the air pouch lavages. Immunized mice demonstrated that the intradermal immunization with *S. aureus* promoted protection in C57BL/6 mice by reducing the bacterial, which was correlated with increased serum concentration of IgG antibodies (IgG1 and IgG2a) against *S*. *aureus*. The increase in IgG2a antibody levels was correlated with a decrease of bacterial load in intradermally immunized C57BL/6 mice, along with production of IL-17A at the inflammation site, as well as IgG1consumption. Similar results were not found in the A/j lineage. In conclusion, a vaccine against *S. aureus* should focus more on the individual characteristics of the host because it is a determinant factor for the success of the immunization.

## Introduction

Bacterial infections occur worldwide and are an important public health problem. Among pathogens, *Staphylococcus aureus* is the main causative agent of bacterial diseases, accounting for millions of deaths every year, mainly for virulence mechanisms and enhanced antimicrobial resistance. *S. aureus* isolates that exhibit resistance to methicillin are referred to as MRSA (methicillin-resistant *S. aureus*) (1,2).

Little is known about the mechanisms of a protective immunity against an infection by *S. aureus*. Some authors believe that the activation of T and B cells by immunization is sufficient to generate a full protection. The inflammatory response is a complex event, with variations that depend on the intrinsic and extrinsic conditions of each individual and of the infectious agent. The variation among humans may be caused by differences among individual characteristics, such as hormonal levels, associated comorbidities, among others. *S. aureus* colonization and invasive disease are not associated with the development of protective immune responses, which is attributable to a large spectrum of immune evasion factors (3,4).

Studies evaluating the protective immunity against *S. aureus* infection are extremely controversial; therefore, new approaches must be undertaken to clarify the mechanisms involved. Animal models of different lineages may simulate infections and treatment approaches that occur in humans, although murine models have characteristics that differentiate them from the human responses to an infection. Nevertheless, the use of different models can raise awareness of the decisive factors in infection control (5).

Mice have several lineages with different immune response profiles. Among them, the C57BL/6 lineage presents a Th1 response profile and the A/j lineage presents a Th2 profile (6). A comparison of the inflammatory response between these 2 models could contribute to the understanding of immune responses against *S. aureus*. Few studies have compared the inflammatory response of two models after a challenge (7,8).

Mice are of great importance due to their great genetic diversity and easy monitoring and manipulation. Several types of studies may be developed with these animals, including a route of infection similar to humans. The intradermal route is described as one of the best ways for the induction of adaptive immunity against pathogens. This route does not lead to tolerance induction and resembles infection in humans (9).

A common route of infection for *S. aureus* is intradermal. In a model of skin immunization with *S. aureus*, a reduction of lesion size and the presence of heavy neutrophilic infiltration was demonstrated. The magnitude of infiltration was correlated with increased production of cytokines, such as IL-17, and chemokines associated with the chemotaxis of polymorphonuclear leukocytes (10,11). Specific antigens for Th17 cells can improve neutrophil performance with the generation of antibodies for opsonization of staphylococcal strains. Th17 cells are currently being studied in generation of forward protection to infections, particularly with the production of interleukin 17 by CD4^+^ T cells in murine models. However, the requirements for the properties and the differentiation of human Th17 cells and cells in animal models induced by pathogens remain poorly understood (12).

Along with Th17 cells, IgG antibodies are critical in defending against this pathogen. Different studies have shown that protective immunity against extracellular gram positive microorganisms is influenced by opsonization. Phagocytosis has the participation of specific antibodies for polysaccharides present in the capsule and is helpful for protection after infection challenge (12,13).

The difficulty of understanding how the immune response generates protection against *S. aureus* infection is perhaps the biggest obstacle to the development of a vaccination Thus, our study aimed to evaluate and compare the immune response in C57BL/6 and A/j mice immunized with a MRSA sample inactivated by heat and subsequently challenged with the same sample of viable MRSA.

## Material and Methods

### MRSA strains

The MRSA ATCC 43300 strain was obtained from the Institute of Biomedical Sciences (ICB) at the Universidade de São Paulo (USP). The samples were stored in a freezer at –80°C. At the time of culture, samples were thawed at room temperature, plated on brain heart infusion (BHI, pH 7.4, HIMEDIA, France), and stored in an incubator for 24 h at 37°C.

### Determination of bacterial inoculum and inactivation by heat

The quantification of bacterial load inoculated in animals was determined by spectrophotometry to obtain the amount of 10^8^ colony forming units (CFU) of MRSA. After quantification, a serial dilution was carried out to obtain 10^6^ CFU, which was inoculated into animals to be immunized. The strains were inactivated by heat (“Heat killed”, 60°C for 30 min) before immunizations. A sample of the inoculum was cultured for confirmation of inactivation.

### Animals

A/j and C57BL/6 mice, aged 6 to 8 weeks, were obtained from the Instituto Multidisciplinar em Saúde, Universidade Federal da Bahia, Campus Anísio Teixeira facilities. The animals were maintained under controlled conditions of temperature with free access to water and food. All procedures involving animals were approved by the Ethics Committee on Animal Use (CEUA) IMS-CAT UFBA.

### Intradermal immunization with inactivated strains of MRSA

The methodology used was based on the literature, with adaptations (14). The experiment was similar and independent in the different lineages of mice. The animals were divided into two groups: control and immunized, with 36 animals in each group. The mice were anesthetized with 50 mg/kg of ketamine and 10 mg/kg of xylazine to perform the intradermal immunizations in the left ear. Each group was immunized three times. The interval between immunizations was 14 days. The animals received 10^6^ CFU of MRSA inactivated by heat in a volume of 10 µL. Control animals received the same volume of sterile saline.

### Challenge with viable MRSA

Challenge was performed using the air pouch model 14 days after the last immunization. At this time, each group was subdivided into two new groups: mice challenged with live strains of MRSA (n=18) and mice challenged with saline (n=18).

### Air pouch model

The preparation of the air pouch was performed as described in the literature, with adaptations (15). Initially, the animals were anesthetized with 50 mg/kg of ketamine and 10 mg/kg of xylazine. The animals were injected with 3 mL of sterile air in the dorsal subcutaneous space. Mice challenged with MRSA received 10^7^ CFU of inoculum suspended in 100 µL of saline. The corresponding controls received the same volume of sterile saline. Subsequently, the animals were euthanized at 6, 12, and 24 h post infection (n=6 per group per time). Thus, after treatment in C57BL/6 and A/j mice, the following groups were formed: immunized with inactivated strain and challenged with live strain (Heat killed/MRSA), immunized with inactivated strain and challenged with saline (Heat killed/Saline), saline injected and challenged with live strain (Saline/MRSA), and saline injected and challenged with saline in the air pouch (Saline/Saline).

Euthanasia was performed by deep anesthesia with administration of ketamine and xylazine at doses of 400 and 40 mg/kg, respectively, intraperitoneally. After euthanasia, blood samples were collected for differential quantification of neutrophil and the serum for quantification of IgG antibodies.

### Inflammatory cell influx

Lavage of the air pouches was carried out with 5 mL of sterile saline and stored at 4°C. Subsequently, the collected material was centrifuged at 300 *g* for 10 min at 4°C. Total cell count was performed in a Neubauer chamber (LojaLab, Brazil). The differential cell counts were performed by citospin in Panotic stained slides and analyzed by light microscopy. The remaining fluid was stored at –80°C for later quantification of cytokines and IgG antibodies.

### Bacterial load

Five microliters of lavage were seeded in BHI plates (pH 7.4, HIMEDIA) and incubated for 24 h at 37°C. The technique used was the pour plate, thus facilitating the quantitation of colonies formed after culture. The CFU quantification was performed with the aid of a colony counter (CP-600 Plus, Phoenix, Brazil).

### Quantification of inflammatory cytokines in the air pouch lavage

The supernatant from the air pouch lavages was used to determine TNF-α, IL-1β or IL-17A cytokines by sandwich ELISA using the ELISA Ready- SET-GO¯ kit (Bioscience, USA). A standard curve was obtained and cytokines were calculated according to the manufacturer’s instructions.

### Quantification of the IgG1 and IgG2a against *S. aureus*

Whole bacteria were used to coat ELISA plates in sodium bicarbonate buffer, pH 9.6 for 18 h. Plates were blocked for 1h with assay diluent 1x (Assay Diluent, BD Biosciences). The lavage fluid or diluted serum (1:50 in assay diluent 1x) was added to the plates (100 µL/well). After 2 h of incubation at room temperature, the plates were washed thoroughly (5 times) with phosphate buffered saline (PBS) plus 0.1% Tween 20 (Synth). Anti-IgG1 and Anti-IgG2a conjugate diluted 1:100 in assay diluent 1x (Assay Diluent, BD Biosciences) was added (100 µL/well) and incubated for 1 h. After washing as described earlier, a solution of tetramethylbenzidine (BD Biosciences) was added to the wells and the color change pattern was observed. The stop solution was added (50 µL/well) and the color intensity was measured on a plate reader (Thermoplate, Brazil) at 450 nM.

### Statistical analysis

Statistical analysis was performed using the Kruskal-Wallis test of GraphPadPrism software (version 5.0, GraphPad Program Inc., USA) and Dunn's post-test. We used the Mann-Whitney test for comparisons between groups. Statistical differences were considered significant at P<0.05.

## Results

### Immunized C57BL/6 mice showed higher bacterial clearance in the air pouch

**Figure 1.**
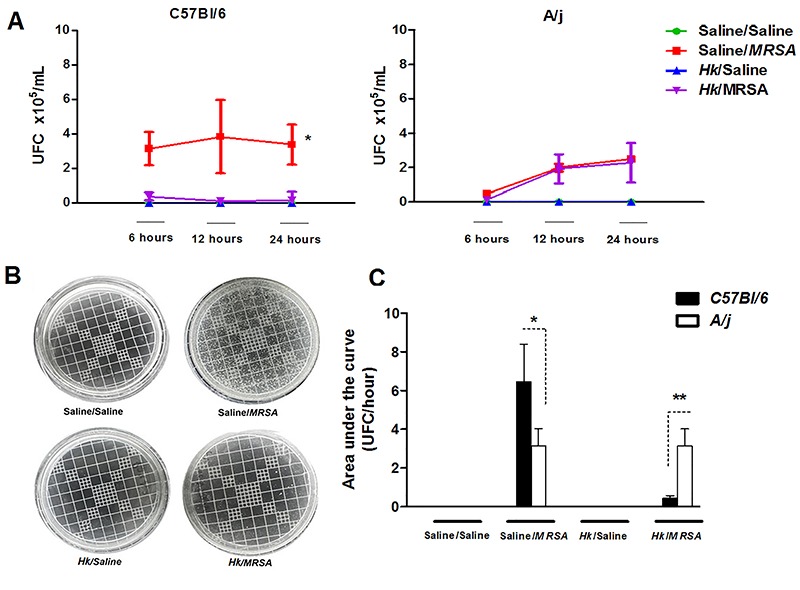
Bacterial load of *Staphylococcus aureus* in the air pouch in C57BL/6 and A/j mice previously immunized with 10^6^ strains of MRSA inactivated by heat and challenged in the air pouch with 10^7^ colony forming units (CFU) live MRSA. *A*, bacterial growth in both animal lineages. *B*, representation of growth in BHI medium for C57BL/6 mice (24 h). *C*, comparison of CFU in the two lineages. Data are reported as means±SD (n=6). BHI: brain heart infusion*;* Hk: Heat killed; MRSA: methicillin-resistant *Staphylococcus aureus*. *P<0.05, **P<0.01 (*A*, Kruskal-Wallis test and Dunn's post-test; *C*, Mann-Whitney test for comparisons between groups).

Figure 1 demonstrates bacterial growth of both strains after 24 h of cultivation. Previously immunized C57BL/6 mice had a lower number of CFU at the different times analyzed. This result was not observed in A/j mice.

### Immunization enhanced IgG2a antibodies production against *S. aureus* in the serum of C57BL/6 mice

**Figure 2.**
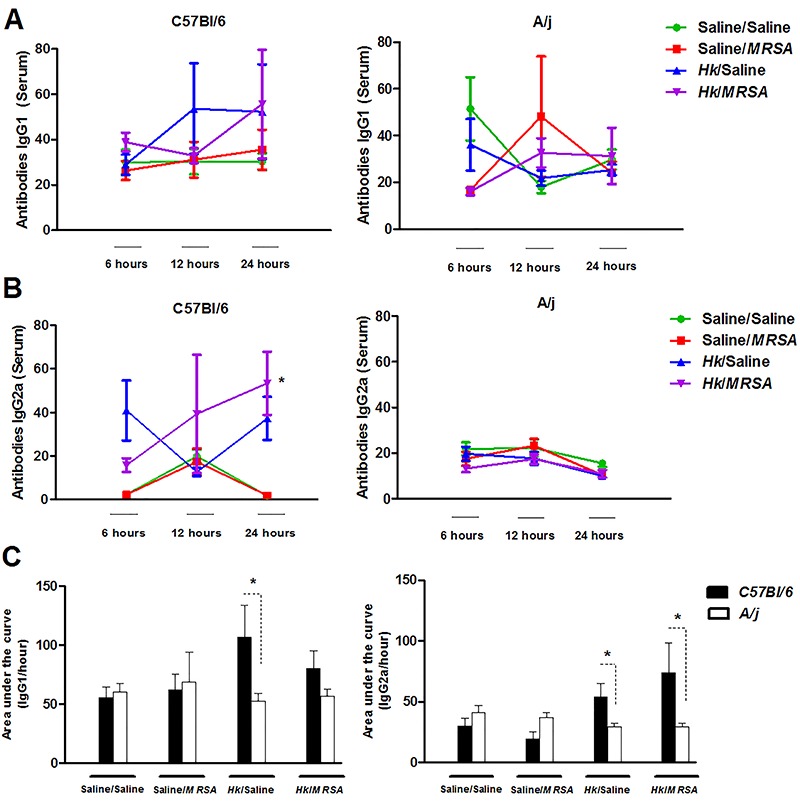
C57BL/6 and A/j mice previously immunized with 10^6^ strains of MRSA inactivated by heat were challenged in the air pouch with 10^7^ colony forming units (CFU) of live MRSA. *A*, Concentration of serum IgG1 antibodies. *B*, concentration of serum IgG2a antibodies. *C*, comparison of antibody concentrations in the two lineages. Data are reported as means±SD (n=6). MRSA: methicillin-resistant *Staphylococcus aureus*; Hk: Heat killed. *P<0.05 (Kruskal-Wallis test and Dunn's post-test *(A* and *B*) and Mann-Whitney test for comparisons between groups (*C*).

In order to assess whether bacterial clearance was related to the production of antibodies, IgG antibodies in serum and from lavage were quantified by ELISA. Figure 2 shows that immunization promoted an enhancement in the titers of IgG2a antibodies in the serum of C57BL/6 animals. Immunized C57BL/6 mice exhibited significantly higher titers of IgG2a antibodies than immunized A/j mice.

### Immunized C57BL/6 mice consumed IgG1 antibodies in the inflammatory environment

**Figure 3.**
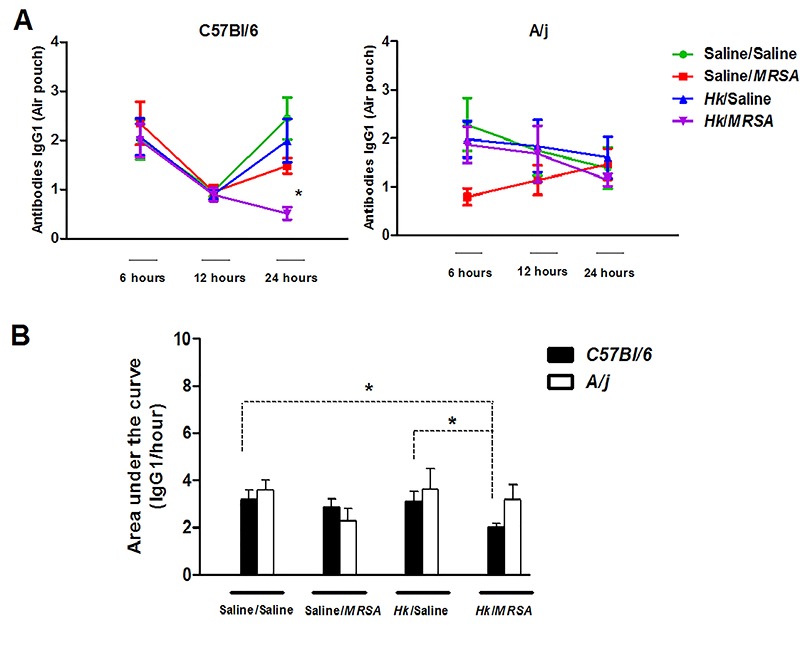
C57BL/6 and A/j mice previously immunized with 10^6^ strains of MRSA inactivated by heat were challenged in the air pouch with 10^7^ colony forming units (CFU) live MRSA. *A*, Concentration of IgG1 in air pouch sample from both lineages. *B*, Comparison of antibody concentrations in the two lineages. Data are reported as means±SD (n=6). MRSA: methicillin-resistant *Staphylococcus aureus*; Hk: Heat killed. *P<0.05 (*A*, Kruskal-Wallis test and Dunn's post-test; *B*, Mann-Whitney test).

To analyze the concentration of antibodies in the infection site, the air pouch lavages were assessed. No difference between the two lineages was observed for IgG1 antibodies. However, there was a decrease in the local concentration of these antibodies 24 h after the challenge only in C57BL/6 mice (Figure 3). There was no significant amount of IgG2a in the analyzed groups.

### C57BL/6 mice showed higher inflammation in the air pouch than A/j mice

**Figure 4.**
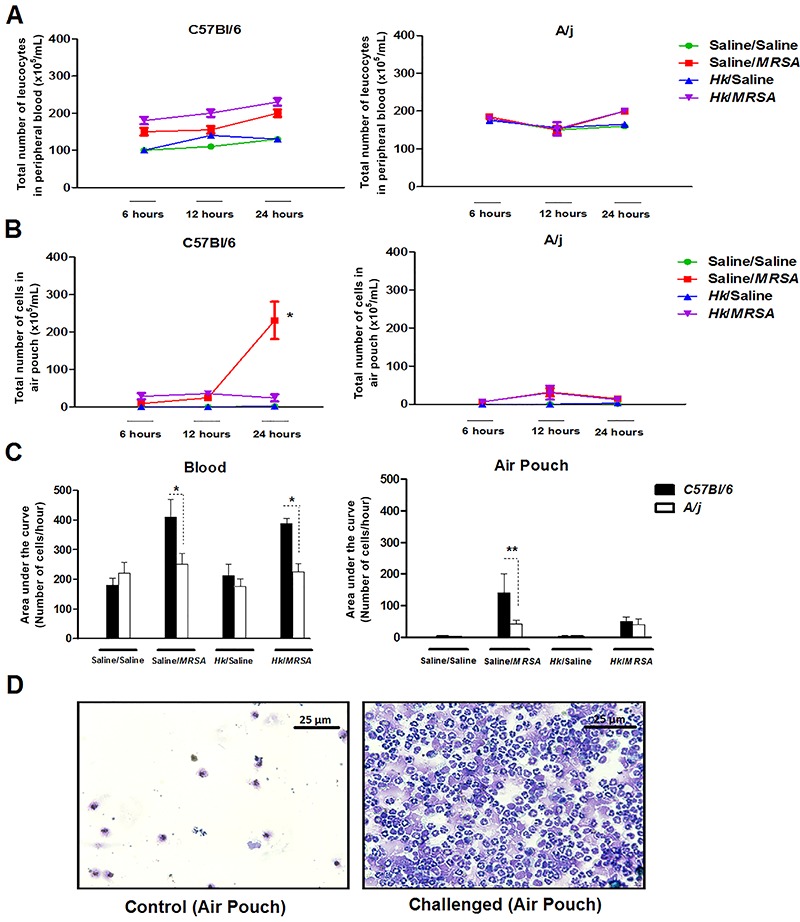
C57BL/6 and A/j mice previously immunized with 10^6^ strains of MRSA inactivated by heat were challenged in the air pouch with 10^7^ colony forming units (CFU) of live MRSA. *A*, Number of leukocytes in peripheral blood. *B*, Number of leukocytes present in the air pouch lavage sample. *C*, Comparison between the two mouse lineages of the amount of leukocytes present in peripheral blood and in the air pouch lavage. *D*, Representation of the inflammatory infiltrate in the air pouch C57BL/6 controls and infected mice (12 h). Bar=25 µM. Data are reported as means±SD. MRSA: methicillin-resistant *Staphylococcus aureus*; Hk: Heat killed. *P<0.05 (Kruskal-Wallis test and Dunn's post-test; Mann-Whitney test for comparisons between groups).

As seen in Figure 4, data demonstrated that infected animals and their respective controls showed no leukocytosis after the challenge at analyzed time points. However, C57BL/6 immunized and MRSA challenged mice had a higher number of leukocytes than A/j mice.

Interestingly, previously immunized C57BL/6 mice had an early recruitment of inflammatory cells and this number was maintained along the follow-up time. Similarly, unimmunized animals showed a later quantitative elevation of recruited cells. Immunized A/j mice and their controls did not differ in cellular recruitment profile in inflammatory environment, and was quantitatively lower when compared with C57BL/6 animals.

**Figure 5.**
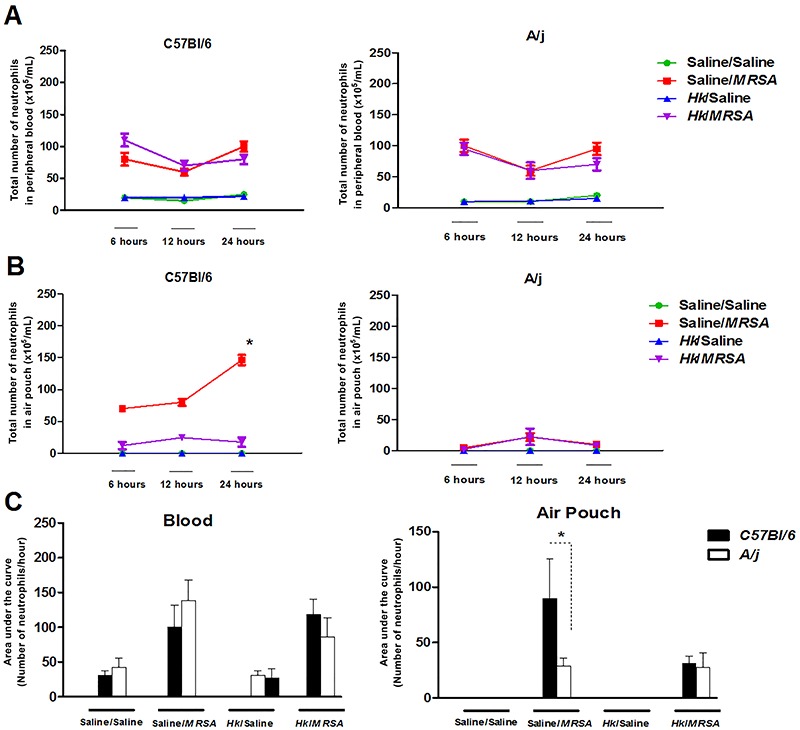
C57BL/6 and A/j mice previously immunized with 10^6^ strains of MRSA inactivated by heat and challenged in the air pouch with 10^7^ colony forming units (CFU) live MRSA. *A*, Total number of neutrophils in the peripheral blood. *B*, Total number of neutrophils present in the air pouch. *C*, Comparison the total number of neutrophils present in peripheral blood and air pouch. Data are reported as means±SD. MRSA: methicillin-resistant *Staphylococcus aureus*; Hk: Heat Killed. *P<0.05 (Kruskal-Wallis test and Dunn's post-test; Mann-Whitney test for comparisons between groups).

The quantification of neutrophils in the peripheral blood and inflammatory environment at all times examined (6, 12, and 24 h) after the challenge in both mouse lineages is shown in Figure 5.

The A/j lineage showed no differences in neutrophil recruitment in the evaluated groups; however, these animals showed lower cell recruitment to the infection site at the analyzed times than C57BL/6 mice.

### Immunized C57BL/6 mice produced IL-17A in the inflammatory site after the challenge

**Figure 6.**
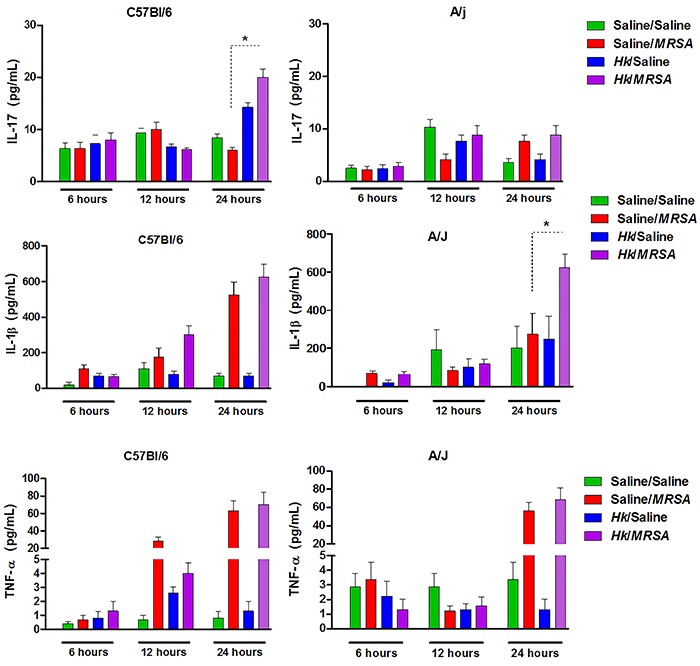
C57BL/6 and A/j mice previously immunized with 10^6^ strains of MRSA inactivated by heat and challenged in the air pouch with 10^7^ colony forming units (CFU) live MRSA. Lavages were collected and evaluated by ELISA for levels of IL-17A, IL-1β and TNF-α (n=6). Data are reported as means±SD. MRSA: methicillin-resistant *Staphylococcus aureus*; Hk: Heat killed. *P<0.05 (Kruskal-Wallis test and Dunn's post-test; Mann-Whitney test for comparisons between groups).

Quantification of cytokines in bone marrow and spleen was performed, and no difference was found in these sites (data not shown). However, it was observed that after 24 h of challenge, immunized C57BL/6 animals showed significant elevations of IL-17A compared to non-immunized challenged C57BL/6 (Figure 6). The analysis of IL-1β pro-inflammatory cytokines and TNF-α by the same technique showed no difference between groups and between the lineages in the analyzed times.

## Discussion

In the infectious process, the generation of an adequate protective response is the main aspect that will determine whether the host will progress to healing or develop disease. The present study showed a correlation between production of IgG antibodies and reduced bacterial load in a murine model of intradermal immunization. In addition, these data were correlated with the increased local production of IL-17. This correlation is important because different studies describe failures after attempting to develop immunizations against *S. aureus*. A reason for this is that this bacterium is present in human microbiota (16). Furthermore, there are significant differences among strains of *S. aureus*, which directly influences the type of inflammatory response against this pathogen and hinders the understanding of the inflammatory process against these bacteria (17).

This is a barrier to a standard understanding of the immune response mediated by antibodies toward this microorganism. Moreover, the pathogenicity and the production of virulence factors are widely described in strains resistant to antibiotics. We analyzed an antibiotic resistant strain, and the air pouch model proved to be effective in assessing inflammation (18). We collected material from the inflammatory environment after evaluation of bacterial load, antibody dosage, cytokines dosage, and local cellular quantification. In addition, it was possible to visualize polymorphonuclear leukocytes (PMNs) in the air pouch. This is important because neutrophils are the main cells in the control of *S. aureus* infections (19).

The lower bacterial load in immunized mice correlated also with lower inflammation in the air pouch. There was a lower amount of neutrophils in the lavage fluid of the immunized C57BL/6 mice in the analyzed time points; however, as mentioned before, even with a smaller amount of PMNs these animals had higher bacterial clearance. This is relevant because the higher the inflammation, the greater the tissue damage that it can cause to the host (19,20). Therefore, the recruitment of cells to the infection site and their activation could be critical for the control of bacterial load. Additionally, intradermal immunization was able to confer systemic immunity, once the challenge occurred at a different body site from the immunization, being an important immunization route for the activation of dendritic cells and consequently a stimulus for the production of antibodies (21).

The higher production of antibodies of the IgG class in immunized C57BL/6 animals is another relevant result because IgG receptors make an important connection between the humoral and cellular immune responses (22). These receptors mediate various biological responses such as phagocytosis, endocytosis, capture and clearance of immunocomplexes, cytotoxicity, and release of inflammatory mediators. These receptors belong to the immunoglobulin superfamily, which has several isoforms. These molecules differ in affinity and specificity for IgG isotypes, cell distribution, intracellular signaling, and molecular weights (23).

In this context, it was observed that previously immunized C57BL/6 animals produced more than one IgG antibody subtype. In this way, it is worth mentioning that within the air pouch, the consumption of IgG1 subtype occurred over the analyzed time (Figure 3A and B). In addition, genetic polymorphism is responsible for variations between individuals, which may explain the fact that A/j animals did not significantly increase antibodies as C57BL/6 animals did at the analyzed time point. The structural and functional diversity of FcγR makes these molecules important targets for immunotherapy (24,25).

In animal studies, protective immunity against extracellular microorganisms, such as gram-positive bacteria, is influenced mainly by opsonization and phagocytosis, with participation of specific antibodies for polysaccharides present in the capsule. This mechanism is an efficient protection after infection challenge (13). Antibodies of the IgG2a class are fundamental in response against *S. aureus*. Different authors have described the participation of IgG2a in immunity against this pathogen (26). The fact that immunized C57BL/6 mice showed protection and decreased bacterial loads in the inflammatory environment could be related to antibodies provided by immunizations. The non-immunized C57BL/6 mice presented higher bacterial loads in their air pouch (Figure 1).

Higher titers of IgG2a are important elements against bacterial structures, such as capsular polysaccharides, teichoic acid, and lipoteichoic acid (13,27). The differences between mouse lineages in the production of antibodies and protection could be related to the interaction between IgG antibodies with proteins of the complement system. For example, IgG1 can bind to C1q and activate the classical complement pathway opsonization, leading to phagocytosis of the *S. aureus* mediated by PMN cells (28).

Studies with other pathogens indicate the importance of the interaction of IgG antibodies with the complement system components such as C5. Such interaction causes improved opsonization and a better targeting of inflammatory processes. It is well known that the A/j mice have a deficiency in complement C5 (29,30). In addition, complement-antibody interaction is deficient in these animals. These data are in accordance with the higher susceptibility of A/j mice to MRSA infection. However, different authors have reported that the complement alone without direct participation of antibodies is not sufficient to cause bacterial clearance of gram positive bacteria by neutrophils and macrophages. Thus, the protection observed in the immunized C57BL/6 mice may be due to a mutual action of complement and antibody of the IgG class (30). Corroborating this fact, non-immunized C57BL/6 mice have the complement system and yet there was no protection in these mice. Other authors report that the deficiency of these components in the A/j model leads to a deregulated response in cytokine production (31).

Another result of this work is the evaluation of IL-17 participation against *S. aureus* infection. There are few reports that address the importance of IL-17A profile after infection by MRSA. Antibodies can enhance the neutrophil response; however, other factors have a strong impact on PMNs activation, such as Th17 cells (11). Among the sites that are colonized by *S. aureus*, the nasal passages are the most common. It has been shown that IL-17 knockout mice are unable to effectively eliminate *S. aureus* (32). Thus, immune responses associated with Th17 could be a strategic method to eliminate persistent infections in humans. Many studies have described that this cytokine is produced by T lymphocytes, but this component is mostly secreted by cells of the innate immunity in inflammatory processes The release of IL-17A by neutrophils has been described by different authors. Mice deficient in IL-17A or IL-17F have proved prone to skin infections caused by gram-positive bacteria such as *S. aureus* (12,33,34).

In one study, administration of neutralizing antibodies to IL-17A was correlated with decreased formation of intra-abdominal abscesses, decreased defense, and exacerbated systemic bacterial infections (11). Corroborating these data, our results showed an enhancement in the local production of IL-17 by immunized C57BL/6 mice 24 h after the challenge. Thus, the inflammatory response promoted by immunization was important for protection in these animals. Different authors have reported that previous exposure to *S. aureus* led to protection inducing IL-17 production. There are few reports that address the importance of IL-17A profile after infectious processes by MRSA (35).

The use of animal models in research is fundamental for the advancement of science since, due to ethical limitations, it is not always possible to conduct studies in humans. Therefore, animal studies have generated findings of enormous impact on the living conditions and longevity of the human being (36). Rodents, especially mice, are the most used animals in research, serving as tools to answer specific questions about human diseases. Thus, we used two strains of mice to evaluate differences between them after being immunized and challenged with *S. aureus*. Altogether, these data can shed light on the understanding of different immune responses among individuals from the same species but with diverse genetic backgrounds (37).

After analyzing the data of this work, it was possible to perceive that even among animal species of the same group, the effectiveness of a vaccine against *S. aureus* will depend on the individual variations. In C57BL/6 animals, different components were produced compared to A/j mice, even though they were from the same species, the same model of infection was used, with equal bacterial load and analyzed in equal time points (7,8).

Mouse strains are divided into lineages that result from mating between siblings (inbred) called isogenic, while colonies from random mating (outbred) are said to be heterogenetic. Each inbred line has a unique gene set, since the consanguineous mating decreases the frequency of heterozygous genotypes, generating a homoallelic state, that is, where there is only one allelic variant present that leads to the fixation of the alleles of that group.

Regarding the other cytokines analyzed, no differences were found between groups. This may be related to the time after the challenge that samples were evaluated. IL-β and TNF-α cytokines were elevated in the air pouch sample from the immunized animals, but had no significant difference compared to groups that did not receive immunization. In *S. aureus* immunization models, cytokines such as IFN-γ had no significant elevation; however, these data are controversial, requiring further studies to elucidate the role of this cytokine after immunization (38).

A/j animals did not show protection after the challenge with live strain and no increase in IgG antibody and IL-17A were observed. On the other hand, the presence of these features was associated with lower bacterial load in immunized C57BL/6 mice. C57BL/6 and A/j mice are widely studied, especially to investigate the genetic control of host immunity to infectious pathogens. In addition, in sepsis experimental models, A/j mice showed increased susceptibility to infections compared to C57BL/6 mice, especially during the acute phase of infection. However, further work must be done for full clarification of Th17 cell activity and humoral response in the control of MRSA infection.
